# When the parasite strikes back: Secondary paraspinal hydatidosis: A case report

**DOI:** 10.1016/j.ijscr.2025.112075

**Published:** 2025-10-16

**Authors:** Faten Limaiem, Mouadh Nefiss, Anis Bousrih, Ramzi Bouzidi

**Affiliations:** a University of Tunis El Manar, Faculty of Medicine of Tunis, Tunisia; b Pathology Department, Hospital Mongi Slim La Marsa, Tunisia; c Department of Orthopedic Surgery, Hospital Mongi Slim La Marsa, Tunisia

**Keywords:** Echinococcosis, Hydatid cyst, Intramuscular, Paraspinal muscle, Pathology, Case report

## Abstract

**Introduction and importance:**

Hydatid disease predominantly involves the liver and lungs, whereas intramuscular localization is exceedingly rare, particularly in the paraspinal region.

**Case presentation:**

A 57-year-old man with surgically treated pulmonary and retroperitoneal hydatid cysts in childhood presented with chronic thoracolumbar and left intercostal radicular pain. Imaging showed a multiloculated cystic lesion in the left paravertebral muscles at D11–D12 and D12–L1 with extension into the corresponding neural foramina. He underwent surgical excision and histopathology confirmed *Echinococcus granulosus* infection. Postoperative management included albendazole therapy. At 18 months, MRI showed no recurrence, and the patient was asymptomatic with no radiologic disease. Given the risk of late relapse, long-term follow-up is planned.

**Clinical discussion:**

Muscular hydatidosis can present long after treated visceral disease. Diagnosis depends on clinical suspicion supported by serology and imaging, particularly in endemic regions. Definitive management is meticulous surgical excision with adjunctive antihelminthic therapy to minimize recurrence. Because primary muscular involvement is rare, misdiagnosis is common, necessitating heightened clinical vigilance. In our case, no recurrence was detected at 18 months. However, late relapse remains possible, warranting vigilant long-term surveillance with periodic imaging.

**Conclusion:**

Hydatid disease should be included in the differential for paravertebral masses, even after treated visceral disease. This case highlights the need for long-term surveillance and a multidisciplinary approach. Early recognition and appropriate therapy are essential to prevent complications and recurrence.

## Highlights

• Paraspinal hydatidosis is rare, usually secondary to visceral disease.
• Diagnosis relies on high suspicion, MRI, and serology in endemic regions.
• Radical excision with adjuvant albendazole is the treatment of choice.
• Hydatid disease must be considered in paravertebral cystic lesions.
• Long-term imaging follow-up is crucial to detect recurrence.

## 1 Introduction

Hydatid disease is a parasitic zoonosis caused by the larval stage of *Echinococcus granulosus*, commonly transmitted in areas with close human-livestock-dog interactions. While the liver and lungs are the most frequently affected organs, accounting for approximately 75 % and 10 % of cases respectively, the disease can occasionally disseminate to atypical sites such as the brain, bones, thyroid, and skeletal muscles [1,2]. Primary intramuscular hydatid cysts are extremely rare, likely due to the unfavorable physiological environment of muscle tissue, which includes constant movement and high lactic acid concentration [3]. Paraspinal muscle involvement is exceptionally uncommon and remains poorly understood due to limited reported cases. Most muscular presentations are secondary to prior visceral infection, but their diagnosis is often delayed or missed due to non-specific symptoms and a broad differential diagnosis. The rarity of paravertebral involvement, along with its atypical presentation and imaging features, contributes to frequent diagnostic challenges and therapeutic delays. Moreover, recurrence in muscle tissue after apparently successful treatment of visceral hydatidosis is seldom reported [4,5]. We present a rare case of recurrent intramuscular hydatid disease in the paraspinal region of a patient with previously treated pulmonary and retroperitoneal cysts. This report aims to: (1) raise awareness of late musculoskeletal recurrence in endemic settings, (2) describe key radiological features that may aid in diagnosis, and (3) emphasize the importance of including hydatid disease in the differential diagnosis of paravertebral masses, even in patients with a history of prior treatment. This case report has been reported in line with the SCARE checklist [6].

## 2 Case presentation (Table 1)

### 2.1 Patient history and presenting complaint

A 57-year-old man with a remote history of surgically treated pulmonary and retroperitoneal hydatid cysts 49 years earlier presented with an 8-month history of progressively worsening, dull, constant thoracolumbar pain localized to the left paravertebral region. Over the preceding 4 months, the pain had been exacerbated by an associated left intercostal radiculopathy. The pain demonstrated positional variation, worsening with movement and partially relieved by rest, but persisted as a baseline discomfort. Neurological examination revealed no motor weakness. The patient denied any history of trauma, fever, night sweats, or unintentional weight loss. Although he had previously received albendazole therapy for hydatid disease, he acknowledged poor adherence to long-term imaging surveillance.

**Table 1:** Clinical timeline of secondary paraspinal hydatidosis.

Time point	Event	Key findings/interventions
1975	Initial Diagnosis of pulmonary hydatid cyst	Treated with surgical excision and albendazole therapy.
1977	Recurrence: retroperitoneal hydatid cyst	Treated surgically with albendazole.
March 2023	Onset of dorsolumbar pain	Progressive, dull pain in the left paravertebral region (D11-D12)
01-12-2023	Hospital admission	Tender swelling at D11-D12; no neurological deficits
02-12-2023	Diagnostic workup	**CT/MRI**: 45 × 35 mm multiloculated cystic lesion in left paravertebral muscles; **Echinococcus IgG ELISA (+)**, confirming recurrence
05-12-2023	Treatment	Complete surgical excision via paravertebral approach + albendazole (10 mg/kg/day, divided doses) initiated
25-01 2025	1 year follow-up	No recurrence on MRI; complete pain resolution
June 2025	Periodic surveillance	No recurrence on MRI; patient asymptomatic

### 2.2 Physical examination findings

The patient exhibited a normal gait and stable posture. Physical examination revealed a tender, non-erythematous swelling in the left thoracolumbar paravertebral region (D11-L1), with localized tenderness over the spinous processes and adjacent paraspinal muscles. There was no spinal deformity, inflammation, or neurological deficit. Motor strength, sensory function (light touch/pinprick), and deep tendon reflexes were intact, with no evidence of clonus, Babinski sign, or meningeal irritation.

### 2.3 Diagnostic workup

Baseline laboratory investigations revealed a normal complete blood count with no leukocytosis (White Blood Cells: 7.6 × 10^3^/μL) or eosinophilia (Absolute Eosinophil Count: 100/μL). Inflammatory markers were within normal limits, with a C-reactive protein level of 5 mg/L and an erythrocyte sedimentation rate of 7 mm at the first hour. Serologic testing via a qualitative Echinococcus IgG ELISA was strongly positive (Optical Density ratio: 4.5; reference range: <1.1), confirming the diagnosis of recurrent hydatid disease.

**Fig. 1:**
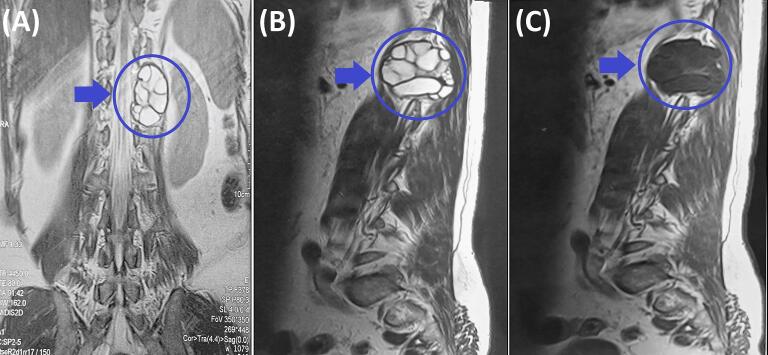
Preoperative thoracolumbar MRI. (A) Coronal T2-weighted image showing a multiloculated cystic lesion (blue arrow and circle) located paravertebrally in the thoracolumbar region from D11 to L1 invading T11-T12 and T12-L1 intervertebral foramen with limited invasion of the spinal canal. (B) Sagittal T1-weighted image demonstrating the cystic lesion's (blue arrow and circle) heterogeneous signal intensity and its proximity to the vertebral column. (C) Sagittal T2-weighted image further characterizing the multiloculated nature of the cystic lesion (blue arrow and circle).

**Fig. 2:**
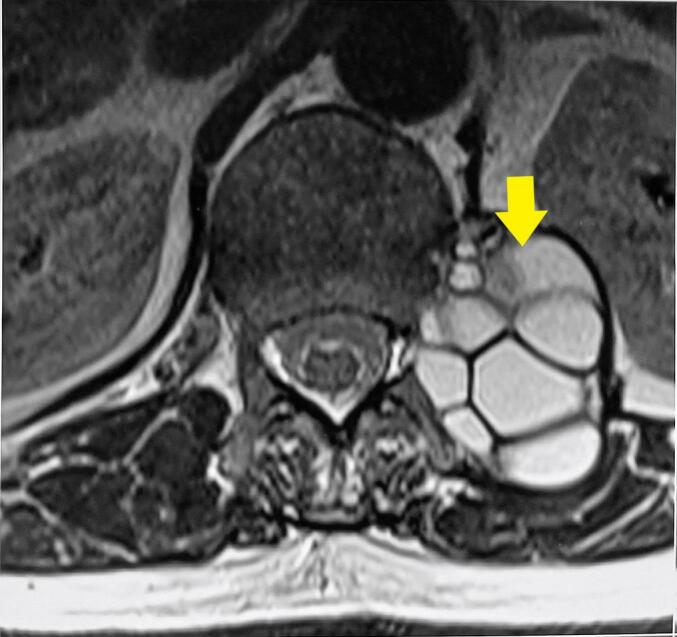
Preoperative thoracolumbar MRI. Axial T2-weighted image revealing the large multiloculated cystic lesion in the left paravertebral region (yellow arrow), with compression and displacement of surrounding structures particularly the left kidney. There is also a close contact the left hemi vertebral body and foramen.

Lumbar computed tomography demonstrated a well-circumscribed, multiloculated cystic lesion measuring 46 × 34 mm in the left paravertebral region at D12–L1, with thin enhancing walls and internal septations after intravenous contrast. Thoracolumbar MRI (Fig. 1, Fig. 2) corroborated a multiseptated cystic mass measuring 45 × 35 mm within the left paravertebral muscles at D11-D12 and D12-L1, extending into the corresponding left neural foramina with limited intraspinal extension. A mild mass effect on the left kidney was noted. Recurrent hydatid cyst was considered the most likely diagnosis. Abscess was unlikely because there were no systemic or laboratory signs of infection and no rim enhancement on imaging. Schwannoma was less probable given the purely cystic, multiseptated morphology without a solid component or “target sign.” Lymphangioma was also improbable owing to its usual cervicothoracic distribution, whereas the paraspinal location with foraminal extension and the patient's history of hydatidosis favored hydatid disease.

### 2.4 Treatment and follow-up

**Fig. 3:**
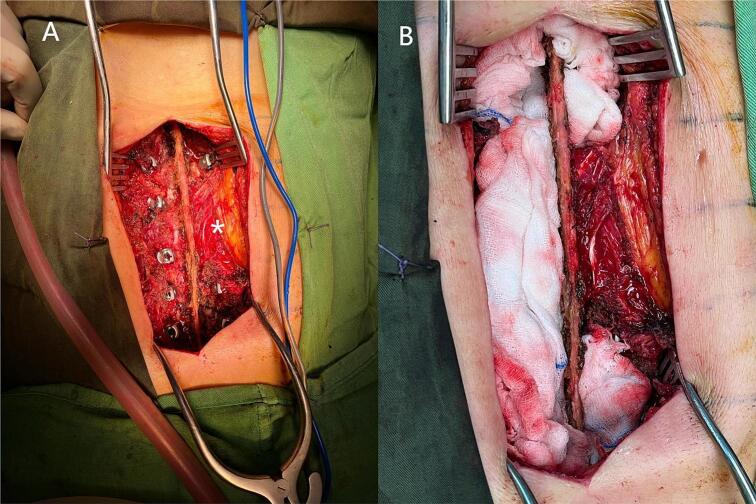
Intraoperative images. (A) Initial surgical exposure with pedicle screw insertion. We preserved part of the muscle and fat all around the cyst which appears bulging on the left side (white asterisk). (B) Protection of the surgical fields with gauze sponges soaked in hypertonic saline for spillage prevention before cyst dissection and excision.

**Fig. 4:**
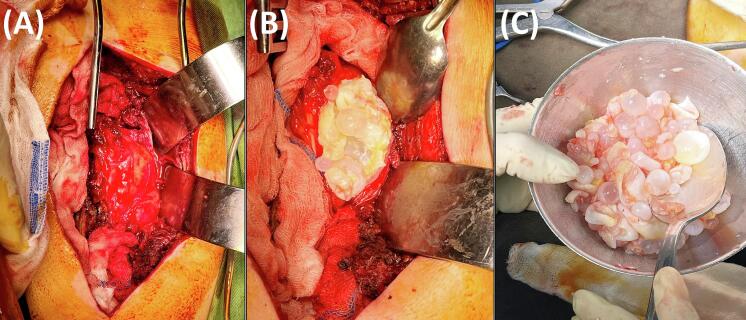
Intraoperative images. (A) Close-up view during surgery demonstrating the multiloculated, whitish, and gelatinous appearance of the hydatid cysts being removed. (B) Excised hydatid cysts collected in a bowl, showcasing their numerous, varied sizes, and characteristic “grape-like” appearance. (C) Final aspect after cyst removal.

**Fig. 5:**
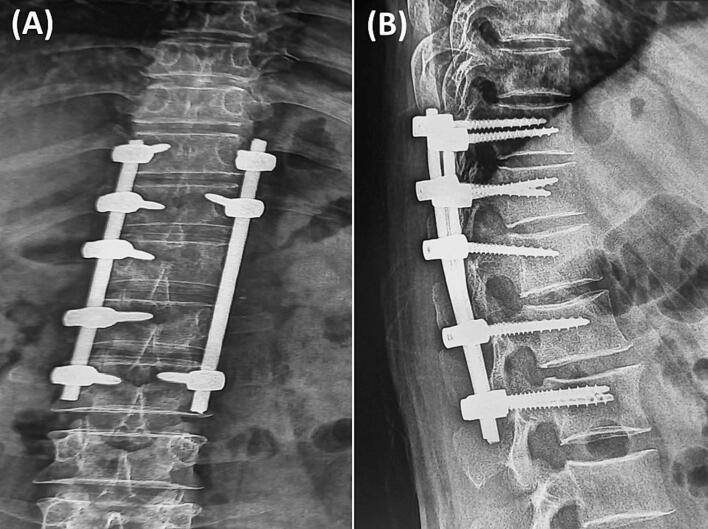
Postoperative thoracolumbar radiographs: (A) anteroposterior and (B) lateral views showing pedicle screw–rod fixation from D10 to L2 for stabilization following hydatid cyst excision.

**Fig. 6:**
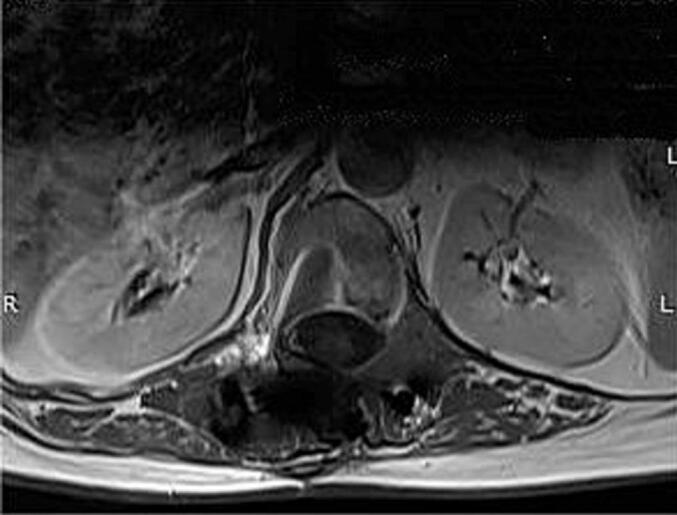
Postoperative thoracolumbar MRI. Axial T2-weighted image at the same level as Fig. 2 showing the complete resection of the cyst and the absence of repression of the left kidney which existed previously.

The patient received no preoperative antihelminthic therapy. Given cyst invasion of the T11–T12 and T12–L1 foramina and the left paramedian spinal canal (Fig. 1A), preplanned posterior instrumentation was indicated, as the approach would require bony resection for neural decompression and cyst excision (laminotomy/facetectomy and costotransversectomy). Because radical en bloc excision was not feasible near critical neural elements, the anticipated risk of recurrence and mechanical destabilization justified posterior fixation to prevent both acute and delayed instability. A midline thoracolumbar incision was made, and left-sided paravertebral muscles were carefully detached to avoid cyst rupture. Bilateral pedicle screws were placed, except at the left T12 and L1 pedicles (Fig. 3A). Screw diameters were 4.5 mm for thoracic and 5.5 mm for lumbar pedicles (Solera, Medtronic). The operative field was protected with gauze sponges soaked in hypertonic saline to prevent spillage (Fig. 3B). Cyst resection, including daughter vesicles, was performed with laminectomy, foraminotomy, and costotransversectomy from T12 to L1 as needed for neural protection and to minimize spillage. The excised material consisted of multiple grape-like cysts of varying sizes (Fig. 4A–C). The postoperative course was uneventful. The patient received albendazole 800 mg/day (400 mg twice daily) for 6 months. Follow-up radiographs confirmed satisfactory coronal and sagittal alignment without screw loosening (Fig. 5). At 18 months, MRI revealed no residual or recurrent disease (Fig. 6). Dorsolumbar pain had resolved completely, and the patient remained neurologically intact at the latest follow-up.

### 2.5 Pathology findings

**Fig. 7:**
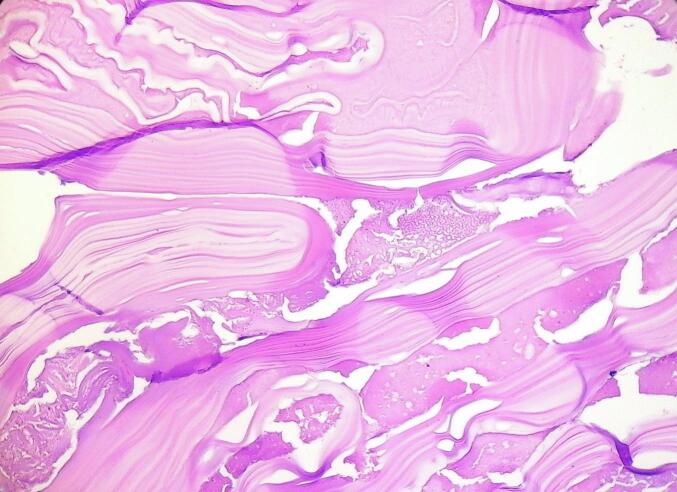
Histopathological examination of excised specimen. Histological section of a hydatid cyst wall displaying laminated acellular layers. Distinctive laminated membrane of the hydatid cyst, characterized by eosinophilic, acellular layers arranged in a concentric, undulating pattern (hematoxylin and eosin, magnification × 200).

Histopathological analysis confirmed the diagnosis by demonstrating a characteristic laminated membrane (Fig. 7) along with the presence of scoleces, diagnostic of *Echinococcus granulosus*. No evidence of malignancy or secondary infection was identified.

## 3 Discussion

Muscular hydatid disease is rare, accounting for only 0.5–5 % of cases [7]. It most often involves the sartorius, quadriceps, adductors, pectoralis major, rectus abdominis, gluteal, and cervical muscles [8,9]. Its rarity is attributed to continuous muscle contraction and high lactic acid concentration, both creating an unfavorable environment for cyst growth [10]. However, muscles with reduced contractility and rich vascularization, such as the cervical group, are more susceptible [11]. Paravertebral muscle involvement is exceptionally uncommon, with only isolated cases reported [5,11]. Musculoskeletal hydatid disease occurs as either secondary infection after hepatic or pulmonary involvement or as primary isolated muscular infection [3,11]. Secondary disease is more frequent, whereas primary muscular hydatidosis remains distinctly uncommon [11,12]. Our case illustrates secondary recurrence after previously treated pulmonary and retroperitoneal hydatidosis, suggesting late dissemination to an unusual intramuscular site despite prior therapy. In contrast, most reported paraspinal cases were primary, with no history of visceral involvement [1,5,[11], [12], [13],^15^,^16^]. In endemic regions, any new paravertebral mass in patients with prior hydatidosis should prompt suspicion of recurrence and justify targeted serologic and imaging evaluation. The most consistent presenting feature in reported cases, including ours, was chronic localized back pain, often associated with a palpable mass [1,5,12,13]. Our patient and others [[1], [2], [3]] lacked systemic manifestations such as fever or weight loss, which may delay diagnosis. Neurological deficits are uncommon in isolated muscular cases [1,2] but become evident when intraspinal extension occurs, sometimes leading to paraplegia [13,14]. Diagnosis relies on both serological testing and imaging. Serological assays detect specific circulating antigens or antibodies, including ELISA for *Echinococcus* IgG (95 % sensitivity, 94 % specificity), immunoelectrophoresis, and hemagglutination tests [15,16]. False-negative results may occur, as reported by Shrestha et al. [1], where the *Echinococcus* IgG test was negative despite a confirmed cyst, likely due to encapsulation and low antigen exposure. Our case, with a positive serology, represents the opposite spectrum. Ultrasonography is the preferred initial modality because it is non-invasive, accessible, and low-cost [9,12]. MRI remains the gold standard for its superior soft-tissue contrast and multiplanar capability [9]. Typical findings include thin-walled cysts with membrane detachment, T1 hypointensity, T2 hyperintensity, absence of enhancement or edema, and clear delineation of surrounding structures. These features help differentiate hydatid cysts from other soft-tissue lesions and reduce the need for invasive procedures. In our case, MRI revealed a multiloculated cystic lesion with septations and no enhancement, consistent with classic features.

Management depends on anatomical location, complications, and patient characteristics [3,4]. For paravertebral or cervical regions, complete surgical excision is the gold standard due to technical difficulty and neurological risk. En-bloc resection offers the best outcome when the cyst can be removed intact [10,11,15,16]. Meticulous technique is essential to prevent rupture and spillage of infectious material. In case of rupture, immediate irrigation with scolicidal solutions such as 10 % aqueous iodine, silver nitrate, or 20 % hypertonic saline is necessary [10,11].

For patients unfit for surgery, the PAIR technique (Percutaneous Aspiration, Infusion of scolicidal agents, and Re-aspiration) under image guidance provides a valuable minimally invasive alternative. Optimal management combines surgery with adjunctive medical therapy. Albendazole remains the cornerstone drug, reducing cyst viability, recurrence, and sometimes cyst size [[9], [10], [11], [12]]. Better outcomes are reported when albendazole is administered both preoperatively and postoperatively, though the optimal duration remains uncertain, ranging from 2 days to 2 months preoperatively and 4 weeks to 36 months postoperatively [9,10,12]. In our case, preoperative albendazole was not administered because of the urgent neurological compromise that required immediate decompression. According to current WHO recommendations, preoperative albendazole (10–15 mg/kg/day for ≥4 weeks) reduces cyst viability and intracystic pressure, thereby minimizing rupture risk and facilitating safer surgical removal. Consistent with Ozsahin et al., such therapy induces germinal membrane degeneration and enhances operative safety, supporting its inclusion in best practice for hepatic and soft-tissue hydatid disease [17]. Postoperatively, the patient received albendazole 800 mg/day for 6 months, consistent with established protocols for secondary muscular hydatidosis. In our case, preoperative albendazole was not administered because of the urgent neurological compromise that required immediate decompression. Although current best practice guidelines recommend a short preoperative course (1–4 weeks) to reduce cyst viability and the risk of intraoperative spillage, such therapy may be omitted when rapid surgical management is necessary. Postoperatively, the patient received albendazole 800 mg/day for 6 months, consistent with established protocols for secondary muscular hydatidosis.

Our case provides an important addition to the limited literature on secondary paraspinal hydatidosis by documenting an exceptionally delayed recurrence nearly five decades after treated visceral disease. Unlike most published cases describing primary muscular involvement, our patient exhibited secondary dissemination without vertebral destruction, emphasizing the unpredictable behavior of hydatid disease. Beyond its rarity, this report highlights critical diagnostic and surgical lessons: the necessity of considering hydatidosis even in atypical paraspinal lesions, the decisive contribution of MRI and serology for accurate identification, and the technical challenges of achieving safe excision near neural structures. The case also illustrates the value of an integrated multidisciplinary strategy, where early recognition and carefully planned surgery can achieve durable disease control despite the complex anatomy of the paraspinal region.

## 4 Conclusion

In conclusion, this case underscores the need to include hydatid disease in the differential diagnosis of paraspinal soft tissue masses, especially in patients with prior visceral involvement and in endemic areas. Although intramuscular hydatidosis is rare, it can manifest long after initial infection and may mimic neoplastic or infectious conditions. Accurate diagnosis relies on imaging, serology, and histopathological confirmation. Complete surgical excision, combined with antiparasitic therapy, offers the best chance of cure. Long-term follow-up is essential to monitor for potential recurrence.

## CRediT authorship contribution statement

Dr. Faten Limaiem: Prepared, organized, wrote, and edited all aspects of the manuscript.
Dr. Mouadh Nefiss, Dr. Anis Bousrih and Pr. Ramzi Bouzidi: Read, edited, and approved the final version of the manuscript. Contributed to data acquisition, analysis, and interpretation. Provided final approval of the manuscript before its submission.

## Consent

Written informed consent was obtained from the patient for publication of this case report and accompanying images. A copy of the written consent is available for review by the Editor-in-Chief of this journal on request.

## Ethical approval

Ethical approval was waived for this case report by the Ethical Committee of Mongi Slim Hospital, La Marsa, Tunisia, as our institution does not require ethical approval for single-patient case reports that are not classified as research.

## Guarantor

Dr. Faten Limaiem.

## Provenance and peer review

Not commissioned, externally peer-reviewed.

## Funding

This research did not receive any specific grant from funding agencies in the public, commercial, or not-for-profit sectors.

## Declaration of competing interest

None declared.
